# Introgression among maternal lineages inferred from complete mitogenomes and molecular dating helps resolve phylogeography of European roe deer

**DOI:** 10.1186/s12862-026-02540-w

**Published:** 2026-06-17

**Authors:** Elena Buzan, Stefan Cidilko, Luka Duniš, Tilen Komel, Boštjan Pokorny, A. J. Mark Hewison, Anna Korzekwa

**Affiliations:** 1 Faculty of Mathematics, Natural Sciences and Information Technologies, University of Primorska, Glagoljaška 8, Koper, 6000 Slovenia; 2 Faculty of Environmental Protection, Trg Mladosti 7, Velenje, 3320 Slovenia; 3 Slovenian Forestry Institute, Večna Pot 2, Ljubljana, 1000 Slovenia; 4 Université de Toulouse, INRAE, CEFS, Castanet-Tolosan, 31326 France; 5 LTSER ZA PYRénées GARonne, Auzeville-Tolosane, France; 6 InLife Institute of Animal Reproduction and Food Research, Biodiversity Protection Research Group, Polish Academy of Sciences, Olsztyn, Poland

**Keywords:** Mitogenome, Introgression, Bayesian molecular dating, Roe deer, Phylogeography

## Abstract

**Background:**

The European roe deer (*Capreolus capreolus*) is one of the most widespread ungulates in Europe, with a phylogeographic structure mainly shaped by Pleistocene glacial cycles and secondary contacts with the Siberian roe deer (*C. pygargus*).

**Methods:**

We sequenced 52 complete mitogenomes of *C. capreolus* from Slovenia, Poland and France, and combined them with 24 publicly available sequences of *C. capreolus* and *C. pygargus*, yielding an alignment of 76 genomes representing 59 haplotypes (42 from *C. capreolus* and 17 from *C. pygargus*). Phylogeographic structure was assessed using a median-joining network, and divergence times were estimated using a time-calibrated Bayesian phylogeny based on mitochondrial coding regions, incorporating published ancient *C. pygargus* mitogenomes. We additionally screened mitochondrial protein-coding genes for selection.

**Results:**

The haplotype network recovered the three major European roe deer clades (Eastern, Central, and Western) and detected Central-clade haplotypes in France. Two Polish haplotypes (Cp9 and Cp10), detected in *C. capreolus*, clustered within the *C. pygargus* mitochondrial lineage, supporting mitochondrial introgression. Time-calibrated phylogenies placed introgressed haplotypes within established *C. pygargus* lineages. Selection analyses provided limited evidence for episodic positive selection restricted to a small number of codons.

**Conclusions:**

Whole mitogenomes improve resolution of roe deer phylogeography and reveal introgressed maternal lineages, while time-calibrated phylogenies and selection tests add evolutionary context for interpreting mtDNA diversity in genus *Capreolus*.

**Supplementary Information:**

The online version contains supplementary material available at https://doi.org/10.1186/s12862-026-02540-w.

## Background

European roe deer (*Capreolus capreolus*) is one of the most abundant and widespread wild ungulates and game species in Europe [1]. Native to the continent, it inhabits a wide range of environments, with a distribution extending from the Iberian Peninsula and the Caucasus Mountains in the south and east, to Fennoscandia in the north, and as far as the Middle East and western Russia [2–4].

The mitochondrial DNA (mtDNA) structure of European roe deer exhibits a complex pattern shaped by Pleistocene isolation in major southern glacial refugia, leading to deep lineage divergence [5–9]. These historical processes have been further shaped by recent extinctions and human-mediated translocations [8]. Phylogenetic analyses conducted by Randi et al. [5] revealed the presence of three major mtDNA clades: Eastern, Central, and Western, corresponding to postglacial recolonization routes of European roe deer from the Balkans, Italy, and the Iberian Peninsula. Although the phylogeographic framework links major lineages to postglacial expansions from the three southern peninsulas (Iberia, Apennines, Balkans), the role of central–southern Italy as a source for recolonisation of European roe deer toward central–northern Europe has been debated, with focused syntheses proposing that large parts of central–northern Europe may have been recolonised mainly from eastern Carpathian refugia, potentially limiting the demographic contribution of Mediterranean peninsulas [5–8]. Subsequent studies have confirmed and refined this phylogeographic structure (6,9–12), although most of the studies relied on partial mtDNA sequences or a limited set of nuclear microsatellite markers. According to previous findings, genetic diversity in European roe deer is unevenly distributed across Europe. Central and eastern clade populations exhibit the highest mtDNA variation, while peripheral populations in Fennoscandia and southern Europe show significantly lower diversity [6, 11]. This phylogeographic mtDNA pattern indicates recolonization from southern refugia following the Last Glacial Maximum [5, 11, 12]. The Eastern clade predominates in Eastern Europe and the Balkans, but also includes haplotypes found in Switzerland, Germany, and Italy [13]. The Western clade is widespread across Western Europe, with a discontinuous range from Portugal to Russia [6]. Most haplotypes within the Eastern and Central clades appear to have expanded during the Weichselian glaciation, particularly around or following the Last Glacial Maximum (~ 26,500–19,000 years ago), while the Western clade likely underwent an earlier expansion during the preceding Eemian interglacial (~ 130,000–115,000 years ago) [6].

One of the key evolutionary processes which has also shaped the genetic landscape of European roe deer is the historical hybridization with the larger-bodied Siberian roe deer (*C. pygargus*), whose current range overlaps with that of the European roe deer in the Volga–Don region [14]. During the Younger Dryas (~ 10,800–10,000 years BP), the two species’ ranges overlapped more broadly, facilitating mtDNA introgression [10, 12]. Although earlier studies attributed the presence of Siberian haplotypes in European roe deer to human-mediated translocations of Siberian roe deer in the 19th and 20th centuries [15–17], recent phylogeographic research has cast doubt on this explanation. Notably, Plis et al. [18] found that introgressed haplotypes in European roe deer are not closely related to those of modern Siberian roe deer, suggesting that much of the introgression likely originated from older, naturally occurring hybridization events, potentially dating back hundreds of thousands of years.

These introgressed Siberian mtDNA lineages are now found across a broad swathe of European roe deer populations in eastern and central Europe, including Poland, Belarus, the Baltic states, Slovakia, Hungary, Ukraine, and European part of Russia [6, 11, 18]. Despite the pronounced signal of introgression in mtDNA, analyses of nuclear microsatellites have not revealed distinct genetic clusters corresponding to Siberian ancestry, likely due to recombination and the gradual dilution of hybridization signals over time [12]. Moreover, complete mitogenome analyses have shown no evidence of positive selection on these introgressed haplotypes, supporting the hypothesis that they are evolutionarily neutral [10]. The widespread occurrence of haplotypes, combined with the phylogenetic complexity and relatively low haplotype diversity, points to an ancient deep and demographically complex process of introgression [12].

The aims of this study were to: (i) estimate the genetic diversity of European roe deer using complete mitochondrial genome sequences; (ii) evaluate whether whole mitogenome analyses support the previously described phylogeographic structure, including the three major clades, and identify introgressed haplotypes across the sampled regions; and (iii) infer time-calibrated phylogenetic relationships to place lineage diversification and introgressed haplotypes in an evolutionary framework. In addition, we assessed signatures of selection in mitochondrial protein-coding genes.

## Materials and methods

### Sampling and DNA sequencing

A total of 52 roe deer muscle tissue samples were collected in Slovenia, Poland, and France, originating from different hunting management districts. All the samples were collected immediately after the harvesting within the hunting season (1 September–31 December) in the period from 2014 to 2024 and were frozen or stored in alcohol until the transportation to the laboratory where they were kept at -80 °C. No animal was shot or otherwise killed for the purposes of this study.

The DNA was extracted with QIAmp DNA Mini kit (Qiagen, Hilden, Germany) following the manufacturer’s instructions. The isolated DNA concentration was determined by the Qubit 3.0 fluorometer using the Broad-Range detection kit (Thermo Fisher Scientific, Waltham, MA USA). The purity of DNA was assessed by measuring the absorbance ratios at 260 nm and 280 nm (A260/A280) for protein contamination and at 260 nm and 230 nm (A260/A230) for other organic compound or salt contamination using Epoch spectrophotometer (BioTek, Winooski, VT, USA).

DNA from 23 samples was sent to Novogene (Beijing, China) for PCR-free library preparation, while libraries from the remaining 29 samples were prepared in-house using the Illumina Tagmentation DNA Prep Kit according to the manufacturer’s instructions (Illumina, San Diego, CA, USA). By combining PCR-free and PCR-based libraries, we achieved both high-fidelity variant detection through minimized amplification bias and improved coverage of challenging genomic regions. Libraries were sequenced in Novogene using the high-throughput Illumina’s NovaSeq X Plus platform, with paired-end sequencing of 150 bp. The data output was approximately 50 gigabases per sample resulting in an 15x coverage.

### Genome assembly and validation

Mitochondrial genome assembly and annotation were conducted using the mitoz all subcommand, which implements the full pipeline from raw read input to annotated mitogenome output in a single step [19]. This tool is specifically designed for organelle genome reconstruction, combining de novo assembly with integrated modules for circularization and annotation.

For most samples, assembly was performed under default parameters, which have been optimized for typical high-quality short-read sequencing datasets. In cases where input data exhibited reduced coverage or lower sequencing quality, parameters were adjusted to improve assembly reliability. Specifically, alternative k-mer lengths (59 and 99) were tested to enhance graph resolution during assembly, and SPAdes was employed as an alternative assembler in place of the default MEGAHIT when initial runs failed to produce complete or contiguous assemblies [20]. These adjustments were applied selectively, ensuring that assembly outcomes remained as consistent as possible across the dataset while also maximizing completeness and accuracy.

The resulting assemblies were automatically annotated within MitoZ, producing gene maps and standardized outputs suitable for phylogenetic genetic analyses. To ensure consistency in comparative analyses, the assembled sequences were subsequently aligned to a uniform starting point using the circlator fixstart subcommand, with the cytochrome oxidase subunit 1 gene specified as the reference starting coordinate. This approach allowed circularized genomes to be standardized across samples, facilitating alignment and downstream analyses.

### Alignment, post processing, and annotation of protein coding genes

Newly assembled mitogenomes that passed the quality controls and those obtained from GenBank were aligned with MAFFT version 7 [21] to the reference *C. pygargus* mitogenome (GenBank ID NC_025271.1) using the FFT-NS-2 alignment strategy and then checked by eye in Jalview version 2.11.5.0 [22]. The sequences were also visually inspected for consistency and to exclude the possible presence of erroneous rearrangements and nuclear pseudogenes [23]. To avoid erroneous hypotheses on homology, all indels, positions with ambiguity in the position of the gaps, and the portion of the control region presenting tandem repeats were excluded. GeSeq [24] was used to identify both protein-coding and tRNA, with the embedded tRNAscan-SE v2.0.5 [25] regions setting the sequence source to circular mtDNA. DNAsp v6 [26] was used to infer the number of haplotypes, haplotype diversity (h) and nucleotide diversity (π), number of polymorphic sites (S), GC content, Tajima’s D and Fu’s (Fs) statistics, for both the whole mitogenome and each individual protein-coding region. The ND6 region was reverse complemented to present the same reading direction as the other protein-coding sequences [27]. DNAsp was additionally used to identify the number of synonymous (SS) and non-synonymous (NSS) sites and to compute the ratio between synonymous and non-synonymous substitutions. The overall mean distance for synonymous substitutions per synonymous site (pS) and non-synonymous substitutions per non-synonymous site (pN) were calculated using Mega 12 [28] according to the Nei-Gojobori method [29] with 1000 bootstraps.

### Detection of selection

Selection acting on mitochondrial protein-coding genes was assessed using codon-based models implemented in HyPhy via the DataMonkey platform [30, 31]. We used FEL to test for pervasive (site-level) selection [32], MEME to detect episodic diversifying selection at individual codons [33] and BUSTED to test for gene-wide evidence of episodic positive selection [34]. Statistical significance was evaluated using the likelihood-ratio framework implemented for each method; sites (FEL/MEME) were considered supported at *p* < 0.10 and genes (BUSTED) at evidence ratio (ER) > 10. Where applicable, we accounted for multiple testing when interpreting site-level results.

### Network analyses

The haplotype network was constructed in PopART software [35] using the median-joining algorithm to visualize relationships among our 35 haplotypes and 16 published in Matosiuk et al. [7] Node sizes in the network were scaled according to haplotype frequency, and mutational steps between haplotypes were indicated by numbers on the connecting lines.

### Bayesian phylogenetic inference and time calibration

Time-calibrated phylogenetic relationships were inferred using coding regions from complete mitochondrial genomes (including four ancient and additional four recent samples from Deng et al. [36], Table S2), resulting in 59 haplotypes for the analysis in BEAST2 v2.7.8 [37]. Substitution-model uncertainty was accommodated using bModelTest [38], rather than selecting a single best-fit model a priori. We applied a strict molecular clock and a constant population size tree prior. Two internal calibration points were used following the *Capreolus* dating framework of Deng et al. [36]: (i) the TMRCA of *Capreolus* and moose (*Alces alces*) with a Normal prior (mean 13.90 Ma; 95% interval 15.89–11.99 Ma), and (ii) the divergence between *Capreolus* and water deer (*Hydropotes inermis*) *with* a Normal prior (mean 9.48 Ma; 95% interval 11.31–7.76 Ma). Markov chain Monte Carlo (MCMC) analyses were run for 50,000,000 iterations, sampling every 1,000 steps. Convergence and effective sample sizes were assessed in Tracer [39], discarding the first 10% as burn-in. The maximum clade credibility (MCC) tree was summarized using TreeAnnotator [37] and visualized in FigTree 1.4.4 [40], then edited in Inkscape v 1.4.3 (https://inkscape.org The geographical locations of the samples (Figure S1) were shown using ArcGIS Pro version 3.6.1 (ESRI Inc.).

## Results

### Diversity of capreolus mitogenome

After quality control screening, we obtained 52 newly sequenced complete mitogenomes of European roe deer, representing 35 haplotypes (33 belong to *C. capreolus* and two to *C. pygargus*; ENA project accession number: PRJEB105452). These sequences exhibited the typical features of mammalian mitochondrial genomes, including incomplete stop codons, overlapping coding regions, and variable start codons [41]. The new sequences were combined with the 16 previously published mitochondrial genomes from [7] (8 belong to *C. capreolus* and 8 to *C. pygargus*; GenBank accession numbers: KJ681480–KJ681495), resulting in a final alignment of 68 sequences with a length of 16,357 bp. After the removal of ambiguous positions, alignment gaps, and the tandem repeat region within the control region, the final alignment comprised 1,291 variable sites (7.89%), including 1,043 parsimony-informative sites (6.38%) and 248 singleton sites (1.52%).

Overall sequences showed high haplotype diversity (h = 0.989; SD = 0.005) and low nucleotide diversity (π = 0.013; SD = 0.002), with considerable variability between the two species, which may be a consequence of the low number of *C. pygargus* sequences included in the analysis (Table 1). A similar difference between *C. capreolus* and *C. pygargus* sequences was observed in the variability of the coding regions, with *C. pygargus* sequences showing in general lower nucleotide diversity (Table S3). However, the GC content and the ratio between synonymous and non-synonymous substitutions were generally concordant between the two sets of haplotypes (Table S3). The dN/dS ratio was higher for *C. capreolus* than for *C. pygargus* haplotypes.

In European roe deer, a total of 41 *C. capreolus* haplotypes were detected, with high haplotype diversity (Hd = 0.984 ± 0.007), but low nucleotide diversity (π = 0.004 ± 0.001). Similar patterns were observed within each clade, with haplotype diversity ranging from 1.000 in the Western clade to 0.977 in the Central clade, and 0.933 in the Eastern clade, respectively. Tajima’s D values were negative overall and across all *C. capreolus* clades (–0.702 to − 1.544), although statistically not significant. Tajima’s D values for *C. pygargus* sequences were positive, however also not statistically significant. Fu’s Fs values were positive for all *C. capreolus* clades; however, none of the values was statistically significant. Therefore, Fu’s Fs does not provide support for demographic expansion in our dataset.

**Table 1:** Genetic diversity of mtDNA of the European and Siberian roe deer haplotypes

	*N*	*P*	h	Hd (SD)	π (SD)	Tajima’s D	Fu’s Fs
Overall (*n* = 68)	68	1291	51	0.989 (0.005)	0.013 (0.002)	-0.702	6.483
*C. pygargus*	10	502	10	1.000 (0.045)	0.013 (0.002)	0.866	-0.756
*C. capreolus*	58	486	41	0.984 (0.007)	0.004 (< 0.001)	-1.470	0.349
Eastern clade	10	82	8	0.933 (0.074)	0.001 (< 0.001)	-1.471	1.438
Central clade	45	233	30	0.977 (0.010)	0.002 (< 0.001)	-1.544	1.214
Western clade	3	52	3	1.000 (0.272)	0.002 (< 0.001)	/	2.428

N = number of sequences, h = number of haplotypes, P = number of polymorphic sites, π = nucleotide diversity, Hd = haplotype diversity, SD = standard deviation

The analysis was conducted according to haplotypes, due to the presence of Siberian roe deer haplotypes in European roe deer. Significance of Fu’s Fs and Tajima’s D values was assessed by coalescent simulations, however, neither statistic was significant

### Positive selection

Tests for selection using HyPhy/Datamonkey were first applied to the combined dataset (both species together) and then repeated for each species separately (Table S4); however, results for *C. pygargus* should be interpreted cautiously due to the small sample size for this species (Table S1 and S2). In the combined analysis, results indicated limited, site-specific episodic positive selection rather than widespread pervasive selection across mitochondrial protein-coding genes. The MEME analysis detected the strongest evidence for episodic selection at ND6 codon 95 (*p* = 0.009), with additional weaker or marginal signals across ND2, COX2, ATP8, ND3, ND4, ND5, and CYTB. Consistent with this, FEL identified little evidence for pervasive selection, with only ND6 codon 95 showing borderline support (*p* = 0.099). At the gene level, BUSTED suggested gene-wide episodic selection signals in several genes (notably ND2, ATP8, ATP6, ND4, ND6, and CYTB), with the strongest statistical support for ND6 (LR = 59.2), a subunit of Complex I. These genes encode core subunits of the oxidative phosphorylation complexes responsible for cellular energy production, so selection acting on them is consistent with adaptation of metabolic and thermogenic capacity, for example in response to differing climatic or energetic demands. In species-level analyses, *C. capreolus* showed only near-threshold MEME signals at several codons, whereas *C. pygargus* retained support at ND2 codon 237 (*p* = 0.048) and a weaker signal at ATP6 codon 30 (*p* = 0.098), pointing to a small number of specific amino acid positions that may have been targets of adaptive change in this species.

### Phylogenetic relationships and divergence-time estimation

**Fig. 1:**
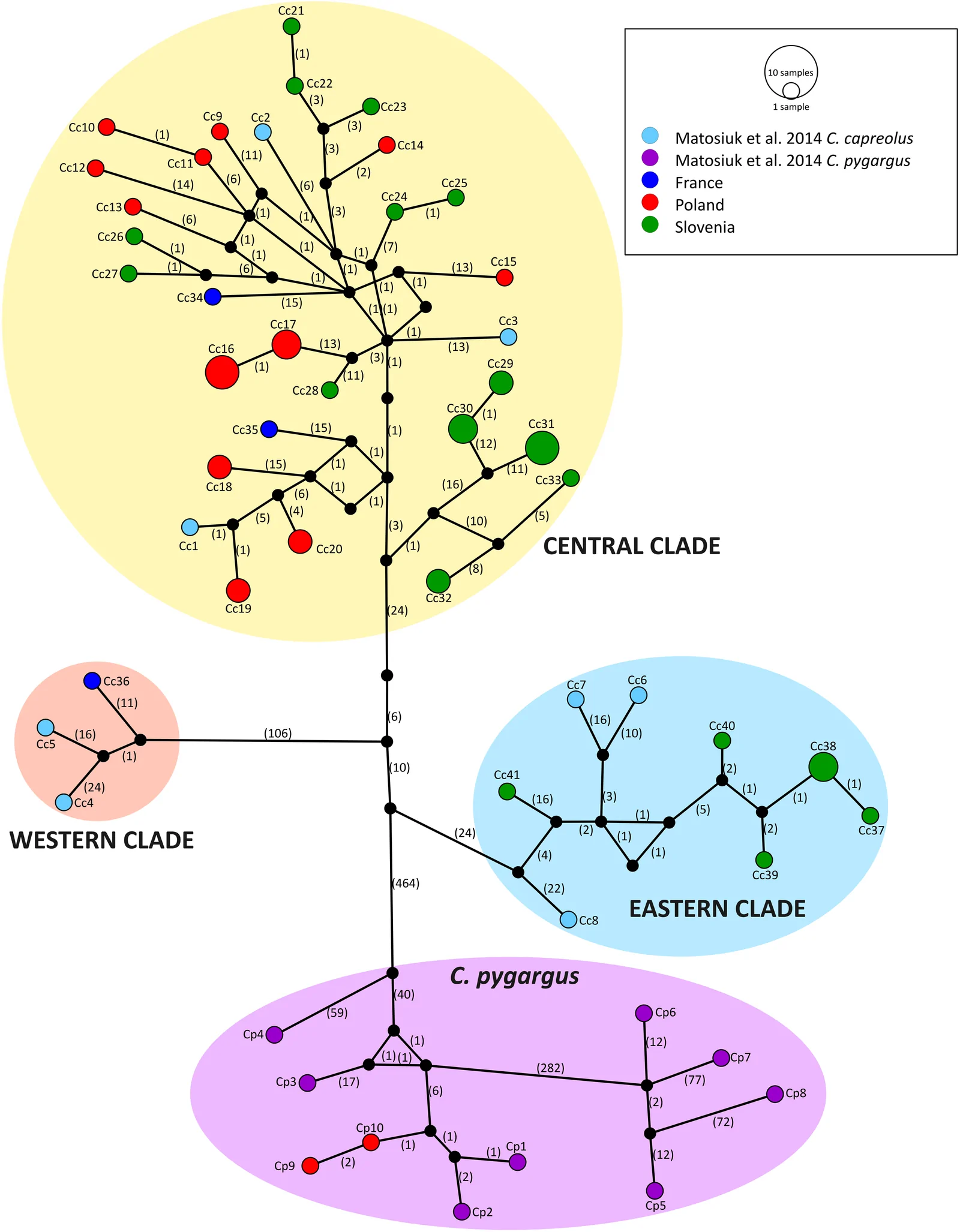
Median-joining haplotype network constructed from 68 mtDNA sequences of *C. capreolus* and *C. pygargus*. Each circle represents a haplotype (labelled accordingly), with circle size proportional to the number of individuals sharing the same haplotype (Table S1 and S2). Numbers in brackets along the connecting lines denote the number of nucleotide substitutions between haplotypes, while black dots represent unsampled or extinct intermediate haplotypes. Circle colours correspond to the country of origin or the reference study from which the samples were obtained

Among the 51 newly detected haplotypes of *C. pygargus* and *C. capreolus* (Fig. 1, Table S1), most belonged to the Central clade, which included haplotypes reported previously by Matosiuk et al. [10] as well as haplotypes from Slovenia and France. The Eastern clade comprised haplotypes from Poland and Slovenia, with Slovenian haplotypes predominating. The Western clade consisted of two previously described haplotypes [10] and a single French sample. Finally, two Polish samples (Cp09 and Cp10) clustered within the *C. pygargus* clade, together with previously reported Russian and Polish haplotypes [1], consistent with mitochondrial introgression.

**Fig. 2:**
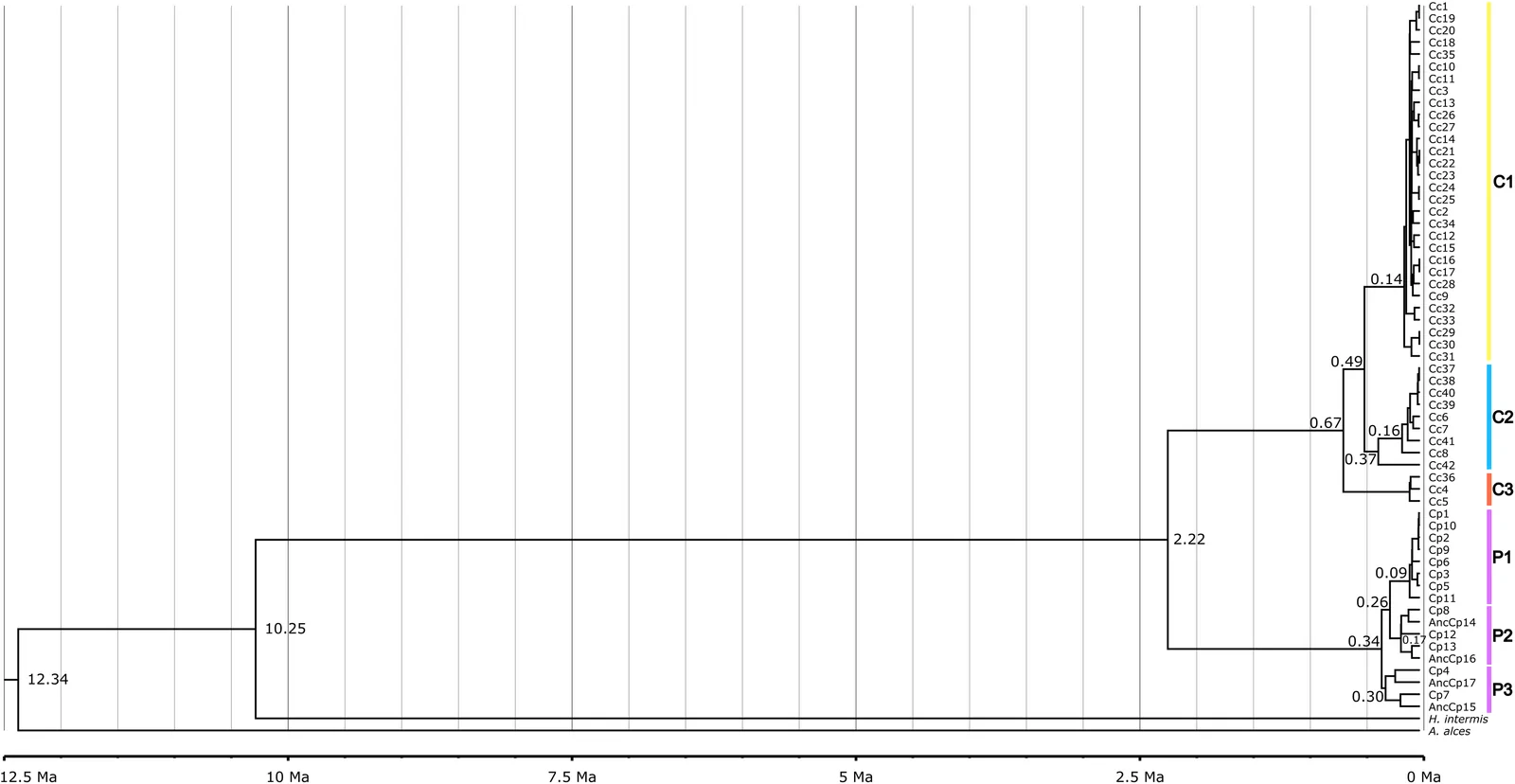
Maximum clade credibility tree of *Capreolus* inferred in BEAST2 from coding regions of complete mitochondrial genomes. Tip labels correspond to haplotype abbreviations (Tables S1 and S2), with ages indicated where applicable. The *C. capreolus* clades correspond to the major European lineages, with C1 representing the Central lineage, C2 the Eastern lineage, and C3 the Western lineage

The time-calibrated tree based on mitochondrial coding regions recovered the expected deep split between *C. capreolus* and *C. pygargus*, dated to ~ 2.22 Ma (95% HPD: 2.82–1.64) (Fig. 2). Within *C. capreolus*, samples clustered into three well-defined clades (C1–C3), with divergence among these lineages occurring in the mid-to-late Pleistocene (e.g., the split leading to C3 vs. the remaining *C. capreolus* clades at ~ 0.67 Ma (95% HPD: 0.87–0.49), followed by additional splits around ~ 0.49 Ma (95% HPD: 0.63–0.35) and ~ 0.14–0.16 Ma (95% HPD: 0.19–0.08). In the *C. pygargus* clade, the topology was structured into three main clades (P1–P3), with P3 occupying the earliest-diverging position, and P1 and P2 forming the remaining more derived diversity. The TMRCA of *C. pygargus* was estimated at ~ 0.34 Ma (95% HPD: 0.43–0.24), and internal diversification within *C. capreolus* clade occurred mainly during the late Middle to Late Pleistocene (e.g., nodes around ~ 0.30 Ma (95% HPD: 0.40–0.21), ~ 0.17 Ma (95% HPD: 0.22–0.11), and ~ 0.09 Ma (95% HPD: 0.12–0.06)). Our haplotypes Cp9 and Cp10, detected in *C. capreolus*, clustered within the *C. pygargus* mitochondrial clade P1, together with previously published haplotypes (Cp1, Cp2, Cp3, Cp4, and Cp12). Clade P1 diverged from the remaining *C. pygargus* clades at approximately 0.26 Ma (95% HPD: 0.35–0.18). Ancient Siberian roe deer mitogenomes from Deng et al. [36] (AncCp) were distributed across the *C. pygargus* portion of the tree nesting within P2 and P3, supporting the persistence of these mitochondrial clades through time.

## Discussion

While numerous phylogeographic studies of European roe deer have relied on single mitochondrial genes or partial mtDNA fragments, comprehensive analyses based on complete mitochondrial genomes remain scarce [10, 36]. By generating and analysing whole mitogenomes, our study provides the first broad-scale, high-resolution assessment of phylogenetic structure and genetic diversity in this species. This approach refines previous insights based on shorter mtDNA fragments, which consistently revealed a phylogeographic pattern structured into three major clades (Eastern, Central, and Western), corresponding to postglacial recolonization routes from the Balkans, Apennines, and the Iberian Peninsula, respectively [5, 9]. However, the contribution of central–southern Italy to postglacial recolonisation of roe deer toward central–northern Europe has been debated, and alternative scenarios involving a stronger role of eastern Carpathian refugia and secondary contact zones have been proposed [6, 7]. In line with the established phylogeographic structure of roe deer, our dataset includes both Western and Central clade haplotypes among the French samples, consistent with secondary contact and the broad distribution of the Central lineage reported elsewhere [5, 7, 9, 10].

Analyses of complete mitogenomes of European roe deer revealed high haplotype diversity (Hd = 0.984), coupled with low nucleotide diversity (π = 0.004), a pattern consistent with previous studies based on partial mtDNA markers [5, 6, 9–12]. Such a combination of high haplotype diversity, but low nucleotide diversity, indicates a recent demographic expansion. However, Tajima’s D was non-significant in all groups, and Fu’s Fs was also non-significant. Therefore, these tests provide no statistical support for recent demographic expansion. These statistics should be interpreted cautiously, because some groups had small sample sizes, reducing statistical power. Moreover, in mitochondrial genomes, negative Tajima’s D values may also reflect purifying selection rather than demographic processes.

Similar signals have been reported in some other studies of European ungulates, where postglacial expansion from refugia resulted in the retention of numerous new haplotypes, but limited nucleotide divergence, with overlapping distributions of different phylogenetic clades (e.g., roe deer: [10]; red deer (*Cervus elaphus*): [42, 43]; moose (*Alces alces*): [44]). This pattern of genetic diversity may also reflect historical migration waves and secondary contacts between populations, similar to contact zones documented in other mammals across Central and Eastern Europe — e.g., wild boar (*Sus scrofa*) in Central Europe, with three mtDNA clades meeting [45–47]; red deer hybrid zones in the Pyrenees and Central Europe [48].

Selection analyses indicated limited, site-specific signals of episodic positive selection (e.g., strongest MEME support at ND6 codon 95) and little evidence for pervasive selection across mitochondrial genes. This pattern is broadly consistent with Matosiuk et al. [10], who found that roe deer mitogenomes are shaped predominantly by purifying selection and reported no convincing evidence for positive selection associated with *C. pygargus* mtDNA introgression, supporting an interpretation of largely evolutionarily neutral mtDNA exchange.

We also confirmed the presence of two haplotypes belonging to the *C. pygargus* lineage in samples from Poland, supporting earlier evidence of historical introgression of Siberian roe deer into European roe deer [10]. As highlighted by previous studies, these haplotypes likely originate from natural hybridization events during glacial periods [7, 10, 14], rather than, or at least not exclusively from, human-mediated translocations in the 19th and 20th centuries [15, 16]. In line with this, our time-calibrated mitogenome phylogeny places *C. capreolus* individuals carrying *C. pygargus* mtDNA within the established Siberian roe deer mitochondrial lineages (P1–P3), consistent with mitochondrial introgression rather than a consequence of recent introductions. Notably, our newly detected introgressed haplotypes (Cp9 and Cp10) fall within P1, the most recently diverged of the three major *C. pygargus* clades (P1 splitting at ~ 0.26 Ma), suggesting that these introgressed variants derive from a comparatively young donor maternal lineage relative to those falling in the older P2 and P3 lineages. More broadly, the late-Pleistocene timing of diversification within the *C. pygargus* clade is consistent with repeated range shifts and secondary contact during glacial cycles, providing a plausible historical window for hybridization and subsequent spread of introgressed maternal lineages in Europe [36]. However, because mtDNA dates lineage coalescence rather than the introgression event itself, distinguishing ancient from recent introgression will ultimately require whole-genome data analysed with explicit demographic modelling.

The detected mitochondrial introgression and the spatial overlap of lineages have implications for conservation and management. In both species, mitochondrial lineages may reflect historical connectivity and past admixture, but they do not necessarily correspond to current demographic units or management boundaries. Identifying areas where distinct lineages co-occur, as well as regions harbouring introgressed haplotypes, can help guide monitoring and inform decisions related to translocations and the delineation of operational management units. More broadly, our results highlight that introgression may be part of the natural evolutionary history of the genus *Capreolus* and should be considered when interpreting genetic structure in applied contexts. Future work integrating whole-genome data with dense geographic sampling will be essential to quantify the extent and direction of hybridization, distinguish mitochondrial introgression from genome-wide admixture, and resolve the demographic processes shaping contact zones, thereby providing a stronger basis for informed management of roe deer populations.

Overall, our mitogenome-based analyses corroborate the phylogeographic patterns previously inferred from partial mitochondrial DNA sequences by Plis et al. [6, 11] and Niedziałkowska et al. [12], and further support the hypothesis that the complex genetic structure of European roe deer across the continent is shaped by a combination of postglacial expansions, ancient hybridization events, and geographic barriers such as the Alps and the Baltic Sea.

## Conclusions

By using a whole mtDNA genome approach, we documented the presence of Central clade haplotypes of European roe deer in France, which is consistent with the well-established phylogeographic pattern of the species and refines the geographic coverage of this lineage compared with earlier mtDNA studies.

Our findings demonstrate that whole mitogenomes provide a higher-resolution tool for detecting phylogeographic structure and for revealing previously hidden historical processes compared to partial mtDNA sequences. To fully understand the evolutionary history of roe deer, future studies should integrate nuclear genomic data to assess the extent of introgression and to explore potential adaptive processes linked to specific genomic regions.

## Supplementary Information

Below is the link to the electronic supplementary material.

Supplementary Material 1

## Data Availability

Data used in this study is available in the supplementary material. All sequence data submitted to NCBI’s GenBank database is also automatically mirrored in the European Nucleotide Archive project accession number: PRJEB105452.
