# Supplementary material for: Introgression among maternal lineages inferred from complete mitogenomes and molecular dating helps resolve phylogeography of European roe deer
Source: BMC Ecol Evol. 2026 Jun 17;26:79. doi: 10.1186/s12862-026-02540-w (PMC13563815; doi:10.1186/s12862-026-02540-w)
Supplement: Supplementary file 1 [file 12862_2026_2540_MOESM1_ESM.pdf]

Supplementary Materials

Table S1. Basic data on *C. capreolus* and *C. pygargus* haplotypes revealed in the study. The "Haplotype" column shows the haplotype labels following the abbreviations used in (Matosiuk et al., 2014).

Sample ID	Location*	Country	Latitude	Longitude	Haplotype
LME5918	Strzałowo	Poland	53.766	21.451	Cc9
LME4626	Mikołajki	Poland	53.802	21.578	Cc10
LME4633	Mikołajki	Poland	53.802	21.578	Cc11
LME5949	Ciechanów	Poland	52.827	20.707	Cc12
LME5951	Ciechanów	Poland	52.824	20.702	Cc13
LME4615	Ciechanów	Poland	52.871	20.607	Cc14
LME4650	Strzałowo	Poland	53.776	21.441	Cc15
LME4631	Mikołajki	Poland	53.802	21.578	Cc16
LME5927	Mikołajki	Poland	53.764	21.592	Cc16
LME5356	Mikołajki	Poland	53.783	21.554	Cc16
LME5917	Strzałowo	Poland	53.767	21.456	Cc16
LME4635	Spychowo	Poland	53.599	21.343	Cc17
LME4654	Mikołajki	Poland	53.802	21.578	Cc17
LME5930	Mikołajki	Poland	53.779	21.557	Cc17
LME4644	Ciechanów	Poland	53.802	21.578	Cc18
LME5950	Ciechanów	Poland	52.824	20.705	Cc18
LME4617	Ciechanów	Poland	52.871	20.607	Cc19
LME5937	Ciechanów	Poland	52.811	20.592	Cc19
LME5933	Mikołajki	Poland	53.913	21.629	Cc20
LME5926	Mikołajki	Poland	53.764	21.592	Cc20
LME0326	LPN Snežnik Kočevska Reka	Slovenia	45.549	14.806	Cc21
LME4868	LD Oljka (Veliki Vrh)	Slovenia	46.345	15.053	Cc22
LME6033	LPN Fazan Beltinci	Slovenia	46.586	16.179	Cc23
LME0125	LPN Ljubljanski vrh	Slovenia	45.935	14.353	Cc24
LME4452	LD Mozirje (Lepa njiva)	Slovenia	46.372	14.979	Cc25
LME1474	Triglav National Park	Slovenia	46.363	13.808	Cc26
LME6035	LPN Fazan Beltinci	Slovenia	46.586	16.179	Cc27

Sample ID	Location*	Country	Latitude	Longitude	Haplotype
LME4457	LD Oljka (Brezovec)	Slovenia	46.336	15.061	Cc28
LME4448	LD Gračišče	Slovenia	45.444	13.859	Cc29
LME4450	LD Izola	Slovenia	45.522	13.674	Cc29
LME1466	LD Koper	Slovenia	45.588	13.729	Cc30
LME1467	LD Koper	Slovenia	45.543	13.730	Cc30
LME1468	LD Koper	Slovenia	45.588	13.729	Cc30
LME4451	LD Mozirje (Brezje)	Slovenia	46.343	14.938	Cc31
LME1475	Triglav National Park	Slovenia	46.363	13.808	Cc31
LME1476	Triglav National Park	Slovenia	46.363	13.808	Cc31
LME1479	Triglav National Park	Slovenia	46.363	13.808	Cc31
LME4867	LD Oljka (Veliki Vrh)	Slovenia	46.345	15.043	Cc32
LME6127	LD Oljka (Veliki Vrh)	Slovenia	46.316	15.060	Cc32
LME6032	LPN Fazan Beltinci	Slovenia	46.559	16.210	Cc33
LME2609	Palaminy	France	43.197	1.075	Cc34
LME2595	Saint-Gaudens	France	43.148	0.745	Cc35
LME2602	Mondavezan	France	43.260	1.037	Cc36
LME1469	LD Koper	Slovenia	45.561	13.781	Cc37
LME4449	LD Koper (Lazaret)	Slovenia	45.593	13.731	Cc38
LME1478	Triglav National Park	Slovenia	46.363	13.808	Cc38
LME0128	LD Mlinše	Slovenia	46.143	14.869	Cc38
LME0129	LPN Medved	Slovenia	45.622	15.114	Cc39
LME6129	LD Oljka	Slovenia	46.316	15.060	Cc40
LME0322	LPN Snežnik Kocevska Reka	Slovenia	45.639	14.780	Cc41
LME4625	Mikołajki	Poland	53.802	21.578	Cp9
LME5539	Mikołajki	Poland	53.783	21.523	Cp10

** In case of Slovenian samples, the name of the respective hunting ground (prefix LD/LPN) is presented, with the concrete location in parenthesis (when available).*

Table S2. List of mitogenomes used in this study retrieved from GenBank with accession numbers and haplotype labels given in the study.

GenBank ID	Species	Country	Haplotype	Reference
KJ681480	*C. capreolus*	Poland	Cc1	Matosiuk et al. 2014
KJ681481	*C. capreolus*	Poland	Cc2	Matosiuk et al. 2014
KJ681482	*C. capreolus*	Poland	Cc3	Matosiuk et al. 2014
KJ681483	*C. capreolus*	Poland	Cc4	Matosiuk et al. 2014
KJ681484	*C. capreolus*	Poland	Cc5	Matosiuk et al. 2014
KJ681485	*C. capreolus*	Poland	Cc6	Matosiuk et al. 2014
KJ681486	*C. capreolus*	Poland	Cc7	Matosiuk et al. 2014
KJ681487	*C. capreolus*	Poland	Cc8	Matosiuk et al. 2014
JN632610	*C. capreolus*	France	Cc42	Hassanin et al. 2011
KJ681488	*C. capreolus*	Poland	Cp1	Matosiuk et al. 2014
KJ681489	*C. capreolus*	Poland	Cp2	Matosiuk et al. 2014
KJ681490	*C. capreolus*	Poland	Cp3	Matosiuk et al. 2014
KJ681491	*C. capreolus*	Poland	Cp4	Matosiuk et al. 2014
MN485773	*C. capreolus*	China	Cp12	Zhang et al. 2020
KJ681492	*C. pygargus*	Russia	Cp5	Matosiuk et al. 2014
KJ681493	*C. pygargus*	Russia	Cp6	Matosiuk et al. 2014
KJ681494	*C. pygargus*	Russia	Cp7	Matosiuk et al. 2014
KJ681495	*C. pygargus*	Russia	Cp8	Matosiuk et al. 2014
MF497305	*C. pygargus*	Korea	Cp11	Kim et al. 2017
MK795818	*C. pygargus*	China	Cp13	Li et al. 2019
OK041025	*C. pygargus*	China	AncCp14	Deng et al. 2022
OK041026	*C. pygargus*	China	AncCp15	Deng et al. 2022
OK041027	*C. pygargus*	China	AncCp16	Deng et al. 2022
OK041029	*C. pygargus*	China	AncCp17	Deng et al. 2022
MN746502	*Hydropotes inermis*	China		Li et al. 2020
KP405229	*Alces alces*	China		Liu et al. 2016

Table S3. Variability among *Capreolus* haplotypes for each gene.

Gene	Species	N	bp	N haplotypes	GC content	π	dN/dS
ATP6	Overall	68	681	21	0.364	0.011	
	C. capreolus	60	681	16	0.363	0.005	0.069
	C. pygargus	8	681	6	0.367	0.004	0.094
ATP8	Overall	68	171	7	0.292	0.148	
	C. capreolus	60	201	5	0.296	0.006	0.137
	C. pygargus	8	171	3	0.259	0.005	0.038
COX1	Overall	68	1545	23	0.370	0.008	
	C. capreolus	60	1545	19	0.370	0.004	0.007
	C. pygargus	8	1545	5	0.371	0.003	0.000
COX2	Overall	68	684	19	0.362	0.009	
	C. capreolus	60	684	16	0.362	0.005	0.030
	C. pygargus	8	684	4	0.363	0.004	0.000
COX3	Overall	68	785	20	0.401	0.011	
	C. capreolus	60	785	15	0.402	0.005	0.052
	C. pygargus	8	785	6	0.396	0.005	0.000
CYTB	Overall	68	1140	35	0.391	0.014	
	C. capreolus	60	1140	29	0.392	0.008	0.131
	C. pygargus	8	1140	7	0.384	0.007	0.007
ND1	Overall	68	957	24	0.362	0.010	
	C. capreolus	60	957	18	0.361	0.004	0.054
	C. pygargus	8	957	7	0.370	0.004	0.000
ND2	Overall	68	1044	28	0.322	0.018	
	C. capreolus	60	1044	23	0.321	0.010	0.159
	C. pygargus	8	1044	6	0.330	0.004	0.010
ND3	Overall	68	348	17	0.366	0.146	
	C. capreolus	60	357	13	0.365	0.007	0.073
	C. pygargus	8	348	4	0.371	0.003	0.000
ND4	Overall	68	1378	32	0.365	0.013	
	C. capreolus	60	1378	26	0.365	0.006	0.117
	C. pygargus	8	1378	7	0.367	0.007	0.035

ND4L	Overall	68	297	12	0.354	0.017	
	C. capreolus	60	297	8	0.354	0.006	0.008
	C. pygargus	8	297	5	0.353	0.004	0.000
ND5	Overall	68	1821	31	0.346	0.150	
	C. capreolus	60	1821	26	0.346	0.007	0.075
	C. pygargus	8	1830	5	0.345	0.006	0.055
ND6	Overall	68	528	19	0.340	0.013	
	C. capreolus	60	528	15	0.340	0.006	0.173
	C. pygargus	8	528	5	0.347	0.005	0.000

Gene = gene analyses, N = number of samples, bp = base pairs, N haplotypes = number of haplotypes, π = nucleotide diversity, dN/dS = ratio of non-synonymous to synonymous substitutions

Table S4. Summary of site- and gene-level tests for pervasive and episodic selection in mitochondrial protein-coding genes, a) between *C. capreolus* and *C. pygargus*, b) among *C. capreolus*, and c) among *C. pygargus*.

a) *C. capreolus* and *C. pygargus*

Gene	Codon	MEME	FEL	BUSTED
ND2	206	0.087	-	13.738
ND2	237	0.053	-	-
ND2	330	-	-	12.119
COX2	40	0.044	-	-
ATP8	47	0.097	-	11.788
ATP6	30	-	-	11.069
ND3	92	0.049	-	-
ND4	2	-	-	10.895
ND4	379	0.048	-	-
ND4	422	0.064	-	-
ND5	104	0.040	-	-
ND5	344	0.039	-	-
ND6	95	0.009	0.099	59.168
CYTB	10	0.081	-	22.590
CYTB	16	0.090	-	20.411
CYTB	302	0.078	-	-

b) *Capreolus capreolus*

Gene	Codon	MEME
COX2	40	0.084
ND3	92	0.093
ND4L	39	0.075
ND5	344	0.075
ND6	95	0.075
CYTB	342	0.099

c) *Capreolus pygargus*

Gene	Codon	MEME
ND2	237	0.048
ATP6	30	0.098

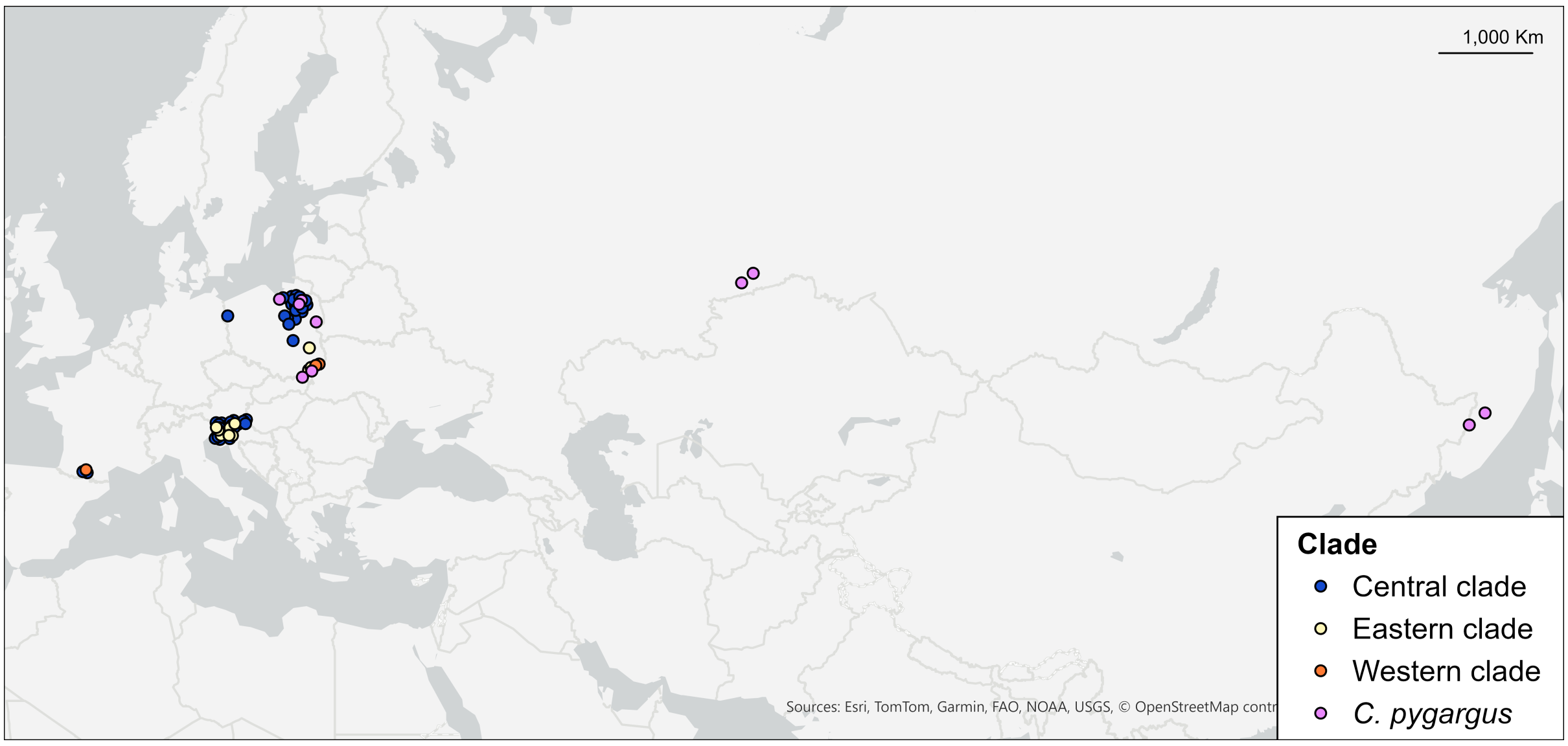

Figure S1. Locations of roe deer samples, included in this study.

References:

Deng, M.-X., Xiao, B., Yuan, J.-X., Hu, J.-M., Kim, K. S., Westbury, M. V., Lai, X.-L., & Sheng, G.-L. (2022). Ancient mitogenomes suggest stable mitochondrial clades of the Siberian roe deer. *Genes*, *13*(1), 114. https://doi.org/10.3390/genes13010114

Hassanin, A., Delsuc, F., Ropiquet, A., Hammer, C., Jansen Van Vuuren, B., Matthee, C., Ruiz-Garcia, M., Catzeflis, F., Areskoug, V., Nguyen, T. T., & Couloux, A. (2011). Pattern and timing of diversification of Cetartiodactyla (Mammalia, Laurasiatheria), as revealed by a comprehensive analysis of mitochondrial genomes. *Comptes Rendus. Biologies*, *335*(1), 32–50. https://doi.org/10.1016/j.crvi.2011.11.002

Kim, H. R., Jeon, M. G., Min, J. H., Kim, H. J., & Park, Y. C. (2017). Complete mitochondrial genome of the roe deer *Capreolus pygargus tianschanicus* (Cervidae) from Korea. *Mitochondrial DNA Part B*, *2*(2), 558–559. https://doi.org/10.1080/23802359.2017.1365645

Kosakovsky Pond, S. L., Poon, A. F. Y., Velazquez, R., Weaver, S., Hepler, N. L., Murrell, B., Shank, S. D., Magalis, B. R., Bouvier, D., Nekrutenko, A., Wisotsky, S., Spielman, S. J., Frost, S. D. W., & Muse, S. V. (2020). HyPhy 2.5—A customizable platform for evolutionary hypothesis testing using phylogenies. *Molecular Biology and Evolution*, *37*(1), 295–299. https://doi.org/10.1093/molbev/msz197

Li, T., Liang, Y.-K., Zhang, J.-J., & Ren, Z.-M. (2019). Complete mitochondrial genome of *Capreolus pygargus* (Cervidae: Capreolinae), a protected and threatened species in China. *Mitochondrial DNA Part B*, *4*(2), 2469–2470. https://doi.org/10.1080/23802359.2019.1638842

Liu, H., & Jiang, G. (2016). Complete mitochondrial genome sequence of Ussurian moose, *Alces alces cameloides*. *Mitochondrial DNA Part A*, *27*(6), 4199–4200. https://doi.org/10.3109/19401736.2015.1022738

Matosiuk, M., Sheremetyeva, I. N., Sheremetyev, I. S., Saveljev, A. P., & Borkowska, A. (2014). Evolutionary neutrality of mt DNA introgression: Evidence from complete mitogenome

analysis in roe deer. *Journal of Evolutionary Biology*, *27*(11), 2483–2494. https://doi.org/10.1111/jeb.12491

Zhang, L., Yang, X., Zhang, H., & Hua, Y. (2020). Phylogenetic relationship of roe deer *Capreolus capreolus mantschurivus* revealed by complete mitochondrial genome. *Mitochondrial DNA Part B*, *5*(2), 1170–1171. https://doi.org/10.1080/23802359.2020.1730731
